# Supplementary material for: Murlentamab, a Low Fucosylated Anti-Müllerian Hormone Type II Receptor (AMHRII) Antibody, Exhibits Anti-Tumor Activity through Tumor-Associated Macrophage Reprogrammation and T Cell Activation
Source: Cancers (Basel). 2021 Apr 13;13(8):1845. doi: 10.3390/cancers13081845 (PMC8070390; doi:10.3390/cancers13081845)
Supplement: Supplementary file 1 [file cancers-13-01845-s001.pdf]

Supplementary Materials: Murlentamab, a Low Fucosylated Anti-Müllerian Hormone Type II Receptor (AMHRII) Antibody, Exhibits Anti-Tumor Activity through Tumor-Associated Macrophage Reprogrammation and T Cell Activation

Mélissa Prat, Marie Salon, Thibault Allain, Olivier Dubreuil, Grégory Noël, Laurence Preisser, Bérangère Jean, Lydie Cassard, Fanny Lemée, Isabelle Tabah-Fish, Bernard Pipy, Pascale Jeannin, Jean-François Prost, Jean-Marc Barret and Agnès Coste

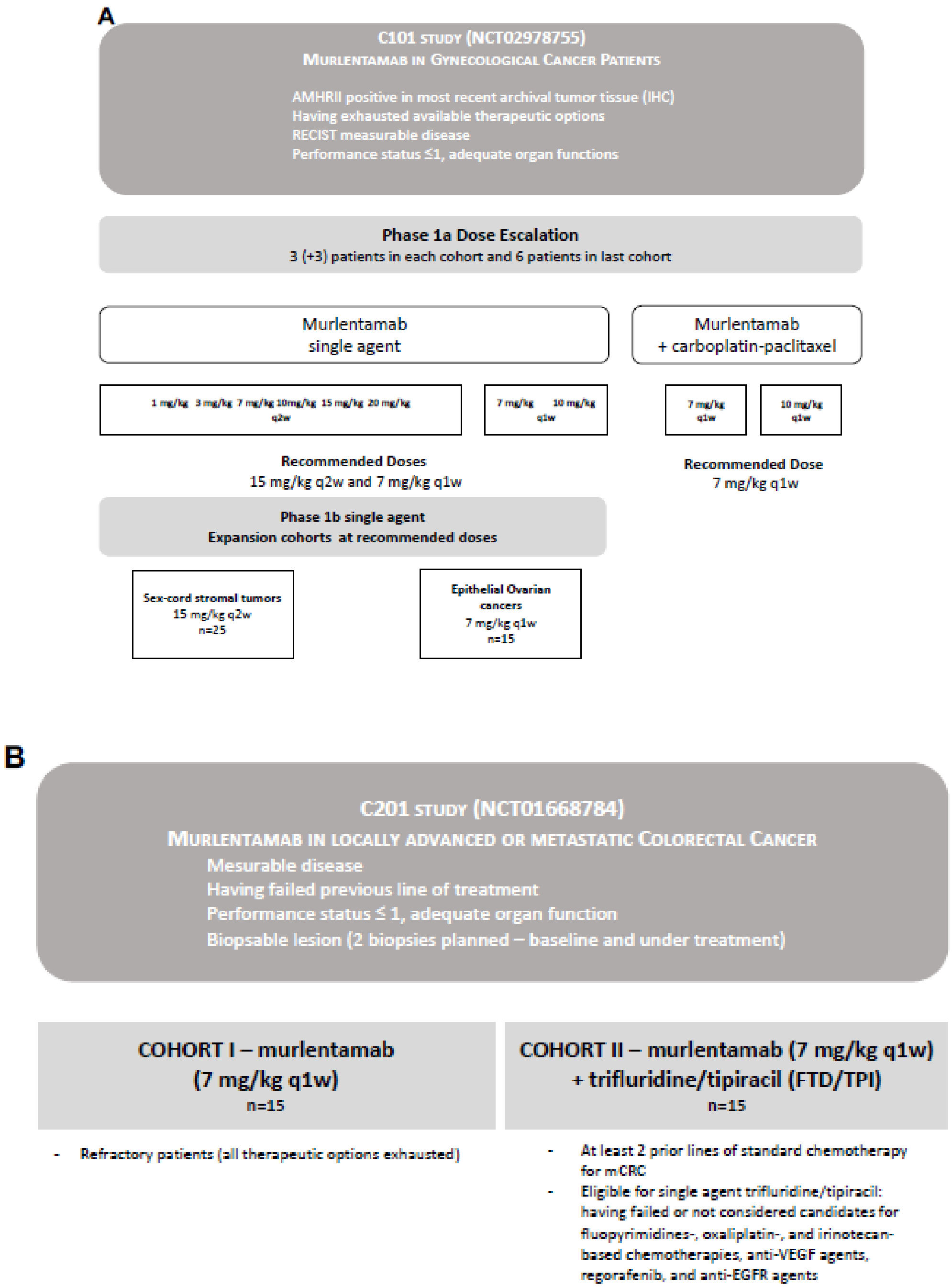

Figure S1. C101 and C201 clinical study design.

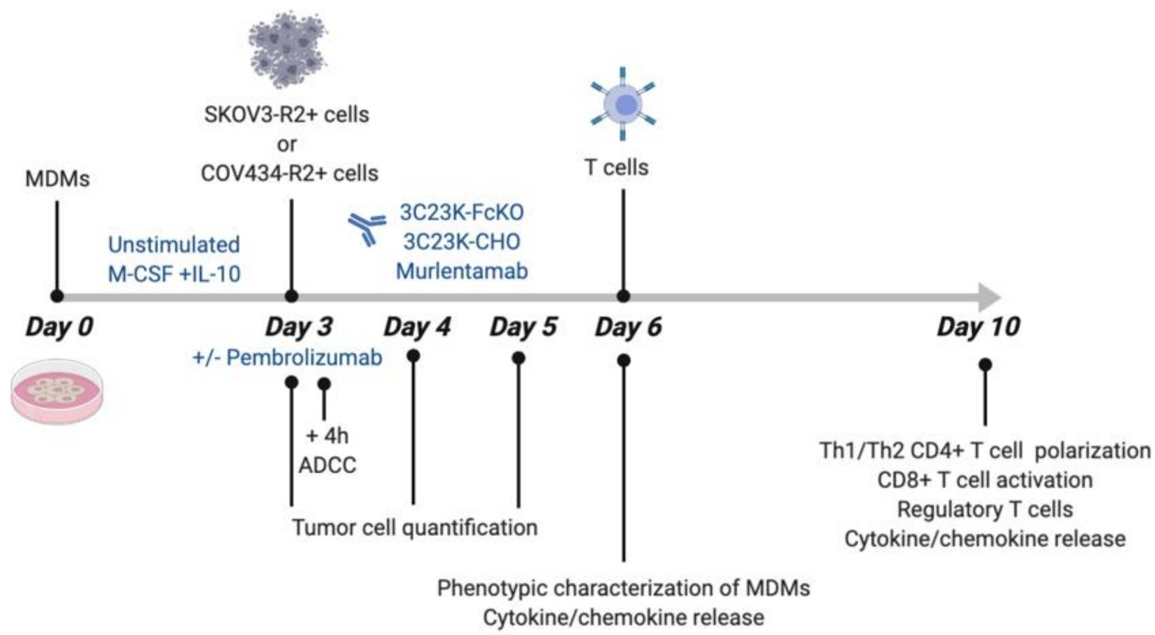

Figure S2. Experimental protocol of the in vitro co-culture system. SKOV3 human ovarian tumor cells modified tostrongly express AMHRII (SKOV3-R2+) were opsonized with murlentamab (also called 3C23K, a low-fucose anti-AMHRII antibody), 3C23K-FcKO (a control antibody mutated in the constant part) or 3C23K-CHO (3C23K normal fucose form) at 10 ug/mL. These opsonized tumor cells were then co-cultured with naïve unstimulated human MDMs (M0) or M-CSF/IL-10- treated human MDMs used to mimic tumor-associated macrophage (TAMs) found in tumor microenvironment. For some experiments, autologous human T cells were added in the co-culture wells. For combo therapy experiments, pembrolizumab (10 ug/mL) was added in the culture everyday until the end of the experiment.

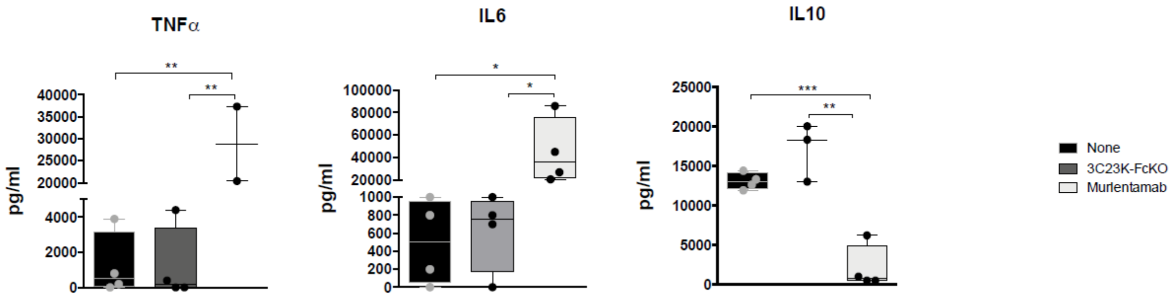

Figure S3. Secretory profile of macrophages cultured with murlentamab. Macrophages were generated by culturing monocytes for 4 days in the presence of M-CSF. These M2-like macrophage were then cultured for 3 days in the absence (None) or presence of immobilized 3C23K-FcKO or murlentamab. Pro- (TNFα and IL6) and anti-inflammatory (IL10) cytokines were quantified in the culture supernatants of LPS-stimulated cells. Results are expressed in ng/ml (boxplots, $n = 4$). *$p < 0.05$; **$p < 0.01$; ***$p < 0.001$; ****$p < 0.0001$. p values were determined using one-way ANOVA analysis followed by Dunnett's multiple comparisons test.

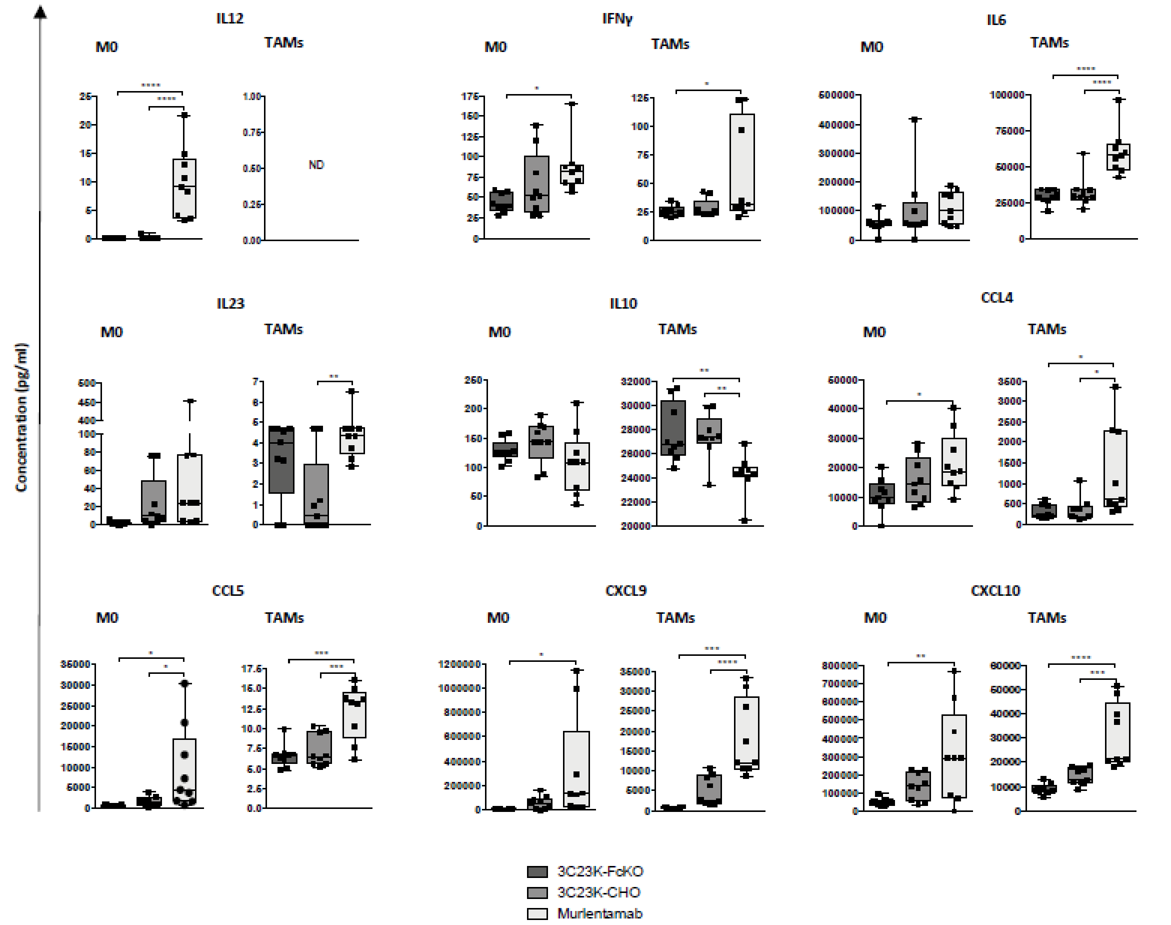

Figure S4. Murlentamab opsonization of SKOV3-R2+ orients naïve macrophages and reprograms TAMs towards a M1-like profile in presence of T cells. SKOV3-R2+ ovarian tumor cells were labeled with different 3C23K antibodies (3C23K-FcKO control, 3C23K-CHO normally fucosylated or murlentamab the low fucosylated form) and cultured in presence of human monocyte-derived macrophages from healthy donors unstimulated (M0) or stimulated with M-CSF and IL-10 (TAMs). After 3 days of co-culture, activated T cells coming from the same healthy donor were added in the culture well for 4 more days. The release of cytokines (IL12, IFNγ, IL6, IL23 and IL10) and chemokines (CCL4, CCL5, CXCL9 and CXCL10) was determined by AlphaLISA. Data shown (boxplots) are the results from three different experiments (performed with three different healthy donors). *$p < 0.05$; **$p < 0.01$; ***$p < 0.001$; ****$p < 0.0001$. p values were determined using one-way ANOVA analysis followed by Dunnett's multiple comparisons test.

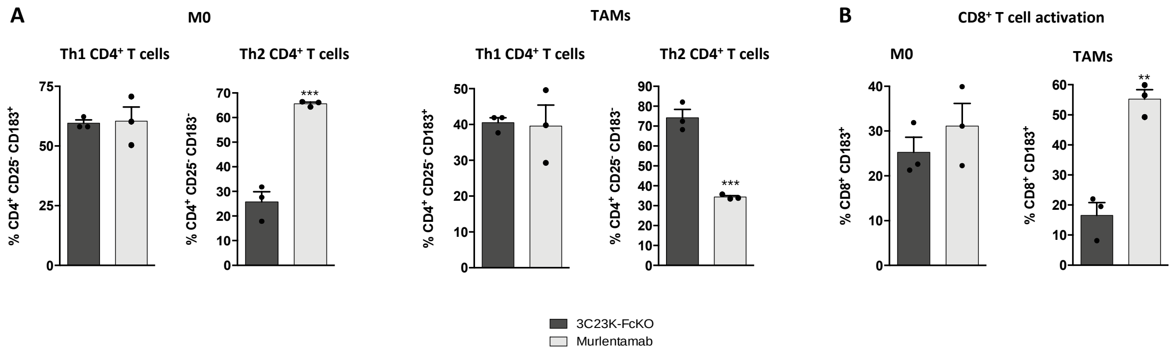

Figure S5. Murlentamab opsonization of COV434-R2+ activates an effective anti-tumor T cell immune response. COV434-R2+ ovarian tumor cells were labeled with different 3C23K antibodies (3C23K-FcKO control or murlentamab the low fucosylated form) and cultured in presence of human monocyte-derived macrophages from healthy donors unstimulated (M0) or stimulated with MCSF and IL-10 (TAMs). After 3 days of co-culture, activated T cells coming from the same healthy donor were added in the culture well for 4 more days. (A) The CD4+ Th1/Th2 polarization profile and (B) the activation of T CD8+ cells were determined by flow cytometry after four days of co-culture. Data

shown (mean ± SEM) are the results from three different experiments (performed with one healthy donors). *$p < 0.05$; **$p < 0.01$; ***$p < 0.001$; ****$p < 0.0001$. p values were determined using one-way ANOVA analysis followed by Dunnett's multiple comparisons test.

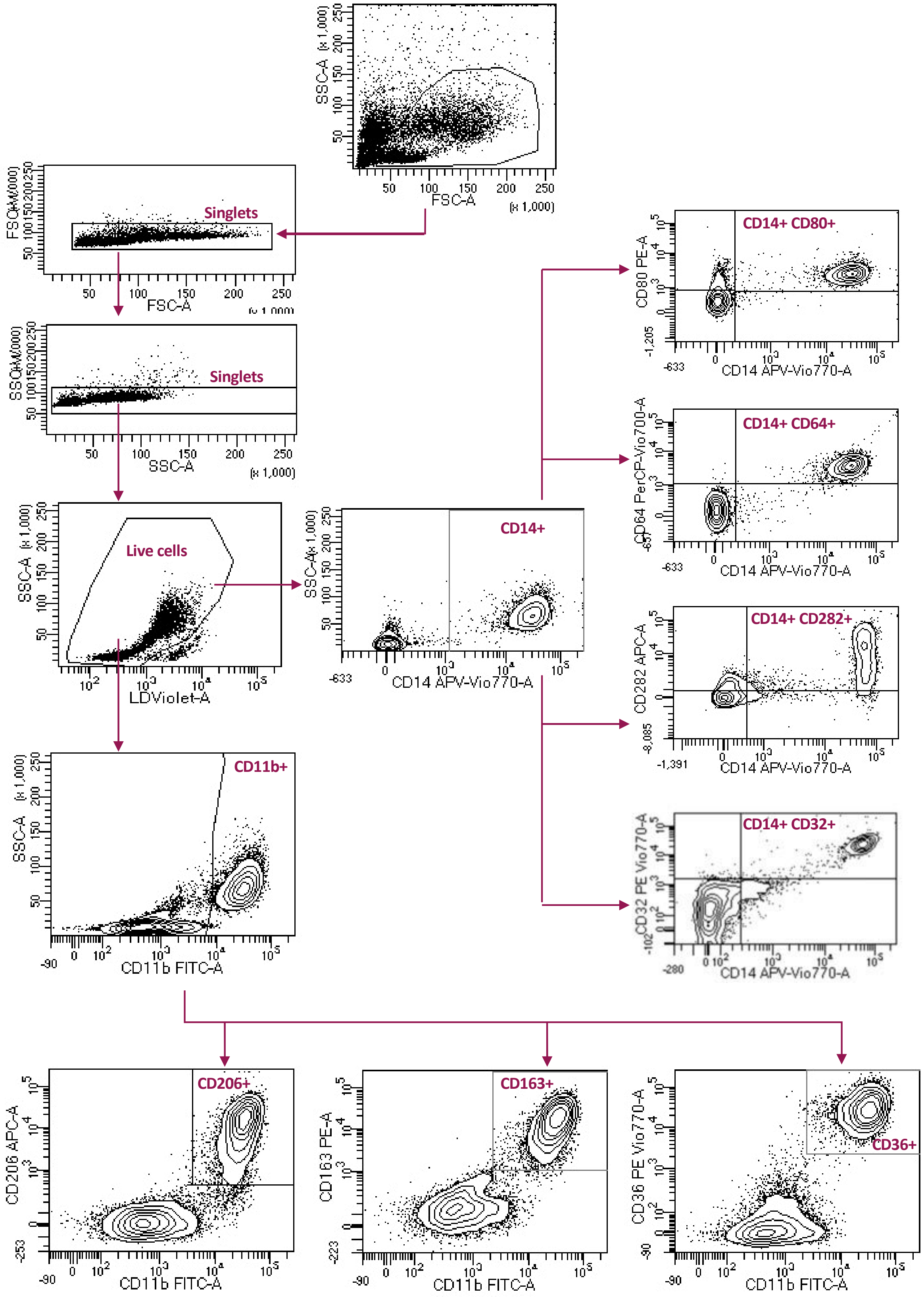

Figure S6. Gating strategies for the analysis of M1/M2 surface receptors at the membrane of human monocytederived macrophages. Forward scatter (FSC) and side scatter (SSC) parameters were used to identify single, viable cells and to eliminate any debris, dead cells and clumps or doublets. Cells

were also stained with a viability dye to make sure to exclude all dead cells from the analysis. CD11b-FITC, CD14-APCVio770, CD32-PEVio770, CD64-PerCPVio770, CD80-PE, CD282-APC, CD163-PE, CD206-APC and CD36-PEVio770 antibodies were used in different staining panels to evaluate the proportions of human monocyte-derived macrophages expressing the CD32, CD64, CD80, CD282, CD163, CD206 and CD36 surface markers as represented in the gating strategies.

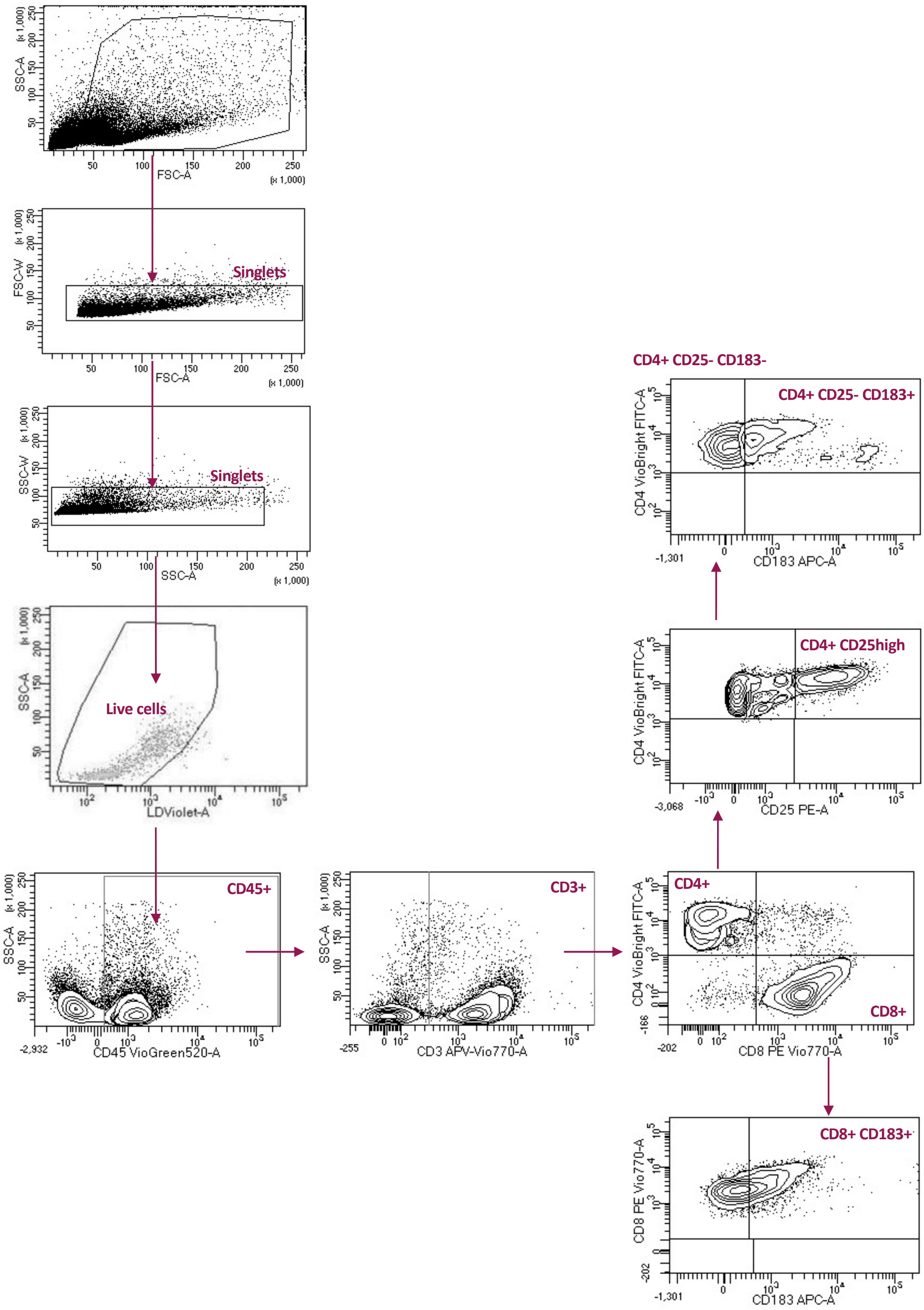

Figure S7. Gating strategies for the analysis of T cells. Forward scatter (FSC) and side scatter (SSC) parameters were used to identify single, viable cells and to eliminate any debris, dead cells and clumps or doublets. Cells were also stained with a viability dye to make sure to exclude all dead cells from the analysis. CD45-VioGreen, CD3-APCVio770, CD8-PEVio770, CD4-Viobright FITC, CD25-PE and CD183-APC antibodies were used to evaluate the proportions of CD8+CD183+, CD4+CD25-CD183-, CD4+CD25high, CD4+CD25-CD183+ T cells as represented in the gating strategies.
